# Effect of metformin on the high‐density lipoprotein proteome in youth with type 1 diabetes

**DOI:** 10.1002/edm2.261

**Published:** 2021-05-09

**Authors:** Evgenia Gourgari, Kristen J. Nadeau, Laura Pyle, Martin P. Playford, Junfeng Ma, Nehal N. Mehta, Alan T. Remaley, Scott M. Gordon

**Affiliations:** ^1^ Division of Pediatric Endocrinology Department of Pediatrics Georgetown University Washington DC USA; ^2^ Division of Pediatric Endocrinology Department of Pediatrics University of Colorado Anschutz Medical Campus Aurora CO USA; ^3^ Department of Biostatistics and Informatics Colorado School of Public Health Aurora CO USA; ^4^ Section of Inflammation and Cardiometabolic Diseases National Heart, Lung, and Blood Institute National Institutes of Health Bethesda MD USA; ^5^ Proteomics and Metabolomics Shared Resource Georgetown University Medical Center Washington DC USA; ^6^ Lipoprotein Metabolism Section National Heart, Lung and Blood Institute National Institutes of Health Bethesda MD USA; ^7^ Saha Cardiovascular Research Center University of Kentucky Lexington KY USA; ^8^ Department of Physiology University of Kentucky Lexington KY USA

**Keywords:** cholesterol efflux, high‐density lipoprotein, metformin, proteomics, type 1 diabetes

## Abstract

**Background:**

Youth with type 1 diabetes (T1D) have normal or elevated High‐Density Lipoprotein Cholesterol (HDL‐C), however, the function of HDL, partly mediated by the HDL proteome, may be impaired. Metformin can be used as an adjunct therapy in youth with T1D, but its effects on the HDL proteome are unknown.

**Objective:**

To determine the effect of metformin on the HDL proteome.

**Subjects:**

Youth (12–20 years old) with T1D who had a BMI > 90th percentile, HbA1c > 8.0% and Tanner stage 5.

**Methods:**

Double‐blinded, placebo‐controlled randomized sub‐study. We examined the effects of metformin (*n* = 25) or placebo (*n* = 10) after 6 months on HDL proteome. Changes in HDL proteins were measured by data‐independent acquisition (DIA) mass spectrometry and compared between treatment groups. As a secondary outcome, associations between proteins of interest and the most studied function of HDL, the cholesterol efflux capacity (CEC), was examined.

**Results:**

The relative abundance of 84 HDL‐associated proteins were measured. Two proteins were significantly affected by metformin treatment, peptidoglycan recognition protein 2 (PGRP2; +23.4%, *p* = .0058) and alpha‐2‐macroglobulin (A2MG; +29.8%, *p* = .049). Metformin did not significantly affect CEC. Changes in affected HDL proteins did not correlate with CEC.

**Conclusions:**

Despite having little effect on HDL‐C, metformin increased PGRP2 and A2MG protein on HDL in youth with T1D, but had no significant effect on CEC. Further studies are needed to understand the impact of PGRP2 and A2MG on other HDL functions.

## Graphical abstract

We hypothesized that metformin could change the protein cargo on HDL and subsequently improve HDL function in youth with T1D. We analysed samples from a large RCT metformin‐placebo trial and we found that despite having little effect on HDL‐C, metformin increased PGRP2 and A2MG protein on HDL in youth with T1D, but had no significant effect on cholesterol efflux. Further studies are needed to understand the impact of PGRP2 and A2MG on other HDL functions.

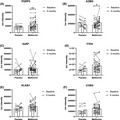

## 1 BACKGROUND

Type 1 diabetes (T1D) is associated with an increased risk of cardiovascular disease (CVD).^1^ This CVD risk is evident even in children with T1D and can manifest as increased carotid intima‐media thickness, increased pulse wave velocity, and decreased brachial artery distensibility.^2^ Among the main risk factors that contribute to CVD risk are dyslipidemia, obesity, insulin resistance and poor glycemic control.^1^ Dyslipidemia has traditionally been defined as low HDL‐cholesterol (HDL‐C), elevated triglycerides (TG), and high LDL‐cholesterol (LDL‐C). The relationship between HDL‐C and CVD risk has been somewhat enigmatic. While epidemiological studies clearly demonstrate an inverse correlation with CVD outcomes, pharmacological manipulation of HDL‐C has repeatedly failed to produce clinical benefit. Recent evidence suggests that the function of HDL is not directly linked to its cholesterol content, therefore the presence of dysfunctional HDL may not be accounted for in the traditional HDL‐C measure.^3^, ^4^, ^5^

The functions of HDL are driven by the protein and lipid components that constitute the complex HDL particle. One of the main functions of HDL is the reverse transport of cholesterol from the arteries to the liver, which initiates with the efflux of cholesterol from peripheral tissues. This is often measured using an *in vitro* assay of cholesterol efflux capacity (CEC) and has been associated with cardiovascular events.^6^ HDL is also involved in atherosclerosis prevention by interfering with coagulation, inflammation, and the complement pathway.^7^ Our group has previously published that youth with T1D have lower CEC compared to healthy controls.^3^ Furthermore, we have shown that youth with T1D have smaller size HDL, which correlated with markers of insulin sensitivity,^3^ and less cholesterol distributed as large buoyant HDL‐C on fast protein liquid chromatography, which correlated with hyperinsulinemic‐euglycemic clamp‐assessed insulin sensitivity.^8^

Based on our previous work, we hypothesized that metformin, which we previously demonstrated improves insulin sensitivity in youth with T1D,^9^, ^10^ could change the protein cargo on HDL and subsequently improve HDL function in youth with T1D. Our objective was to determine the effect of metformin on the HDL proteome in youth with T1D. As a secondary outcome, we examined whether the changes in protein abundance were associated with changes in the CEC, as an indicator of HDL function.

## 2 MATERIALS AND METHODS

This project utilized blood samples that were collected during the “T1D Exchange Clinic Network Metformin RCT Study Group” trial, a double‐blind, placebo‐controlled clinical trial assessing metformin usage as an adjunct therapy in obese adolescents with T1D. The design and the results of the main clinical trial have been previously described.^11^ In summary, this trial was conducted in 26 clinical sites of the T1D Exchange Clinic Network. Eligibility criteria for inclusion in this trial included: age 12–20 years old, diagnosis of T1D, on treatment with insulin for at least 1 year, BMI at or above the 85th percentile based on CDC growth charts, total daily insulin dose of at least 0.8 units/kg/day, HbA1c values at enrollment of 7.5%–10.0% and checking blood glucose at least three times a day. A total of 140 adolescents (aged 12.1–19.6 years old) were enrolled in this study.

### 2.1 Participants

For the current proteomic project, our inclusion criteria included participants with T1D who participated in the “T1D Exchange Clinic Network Metformin RCT Study Group” trial and had a baseline BMI > 90th percentile, HbA1c > 8.0% and were Tanner stage 5. Those criteria were selected in an effort to the minimize potential effects of different stages of pubertal development on protein cargo of HDL. We selected heavier participants and those with suboptimal glycemic control, given the expected lower insulin sensitivity and potentially enhanced benefit from metformin treatment. Of the eligible participants, the core lab randomly selected 25 participants with T1D who were treated with metformin for 6 months and matched them for age and sex with 10 participants with T1D treated with placebo for 6 months.

The T1D Exchange Biobank studies are governed by individual site institutional review boards. Qualifying participants provided consent or assent as age‐appropriate and parents provided consent for participants <18 years old.

### 2.2 Clinical variables

Fasting blood samples were collected for analysis of a lipid profile (total cholesterol, high‐density lipoprotein cholesterol [HDL‐C], low‐density lipoprotein cholesterol [LDL‐C], and triglycerides [TG] at a central laboratory (Northwest Lipid Research Laboratories). HbA1c was measured with an automated glycohemoglobin analyzer (HLC‐723G8, Tosoh Bioscience). Dual‐energy x‐ray absorptiometry (DXA) scans were done to assess % body fat. BMI percentile was calculated using the childs package in r, using the 2000 CDC growth charts as a reference.^12^

### 2.3 Proteomics experiments

The preparation of serum samples for the proteomics experiments was performed as previously described.^13^, ^14^ In brief, (1) purification of HDL from serum using size‐exclusion chromatography; (2) pooling of all the HDL containing fractions; (3) application of lipid‐binding resin to pooled HDL; (4) washing of HDL on the resin in order to remove contaminating proteins; (5) digestion of resin‐bound HDL with trypsin (overnight 37°C); (6) washing of the resin in order to collect tryptic peptides; (7) reduction and carbamidomethylation (DTT and iodoacetamide, respectively); and (8) desalting of samples using ZipTips. The samples were dried for proteomics analysis by using a nano Acquity UPLC coupled with a TripleTOF 6600 mass spectrometer (MS).

As a first step, we established a library of all HDL proteins detectable by MS for the analysis of a sample pool from all the participants (with an equal amount of proteins combined). The pooled sample was analysed in data‐dependent acquisition (DDA) mode. Then, we ran each sample individually via label‐free Sequential Window Acquisition of All Theoretical Mass Spectra (SWATH) data‐independent acquisition (DIA) to quantify each of these proteins in the samples. Specifically, the peptides in each sample were dissolved into 20 µl of 0.1% formic acid. For spectra library generation, the pooled sample was loaded onto a C18 Trap column (Waters Acquity UPLC Symmetry C18 NanoAcquity 10 K 2G V/M, 100 A, 5 μm, 180 μm x 20 mm) at 15 µl/min for 4 min. Peptides were separated with an analytical column (Waters Acquity UPLC M‐Class, peptide BEH C18 column, 300 A, 1.7 μm, 75 μm x 150 mm) which had a controlled temperature at 40°C. The flow rate was 400 nl/min. A 60‐min gradient of buffer A (2% ACN, 0.1% formic acid) and buffer B (0.1% formic acid in ACN) was used for separation: 1% buffer B at 0 min, 5% buffer B at 1 min, 45% buffer B at 35 min, 99% buffer B at 37 min, 99% buffer B at 40 min. The gradient went back to 1% buffer B to equilibrate the column for 20 min. The TripleTOF 6600 mass spectrometer had an ion spray voltage of 2.3 kV, GS1 5 psi, GS2 0, CUR 30 psi and an interface heater temperature of 150°C. The mass spectra were recorded with Analyst TF 1.7 software in the DDA mode. Each cycle consisted of a full scan (m/z 400–1800) and fifty (IDAs; m/z 100–1800) in the high sensitivity mode with a 2+ to 5+ charge state. Rolling collision energy was used. For the SWATH acquisition, each of the samples was injected individually into the same NanoUPLC‐MS/MS system but acquired by repeatedly cycling through 32 consecutive 25‐Da precursor isolation windows, generating time‐resolved fragment ion spectra for all the analytes detectable within the 400–1200 m/z precursor range.

### 2.4 Proteomics data analysis

In order to generate the spectra library, raw mass spectra files after DDA acquisition of the pooled sample were submitted for combined searches using Protein Pilot version 5.0 software (Sciex) utilizing the Paragon and Progroup algorithms^15^ and the integrated false discovery rate (FDR) analysis function.^16^ MS/MS data were searched in the NCBI Homo Sapiens of the Uniprot‐Sprot database containing 20,316 entries (downloaded on June 2, 2015). Carbamidomethylation was set as a fixed modification on cysteine and trypsin was selected as the enzyme. Variable peptide modifications included only methionine (M) oxidation. Other search parameters used were instrument (TripleTOF 6600), ID Focus (Biological modifications), search effort (Thorough), false discovery rate (FDR) analysis (Yes), and user‐modified parameter files (No). The proteins were inferred based on the ProGroupTM algorithm, which is associated with the ProteinPilot software. The detected protein threshold in the software was set at 1% FDR. Peptides were defined as redundant if they had identical amino acid sequence, cleavage site(s), and modification.

For the label‐free SWATH (sequential window acquisition of all theoretical fragment ion spectra) quantification, data from each sample was pre‐processed by PeakView 2.1 (Sciex), with the default settings: (1) Peptide filter: # of peptides per protein: 6; peptide confidence threshold: 99%; # of transitions per peptide: 6; FDR threshold: 1%; (2) Extracted ion chromatogram (XIC) Options: XIC extraction window (min): 5; XIC width (ppm): 75. The retention time was calibrated by selecting 6 peptides with retention time across the whole HPLC gradient. The peak of each transition produced was then manually checked and curated, with only transitions detected in all samples and showing signal to noise (S/N) >10 chosen for peak area calculation. The peptide response was then calculated as the sum of all ion intensity for all curated transitions. The sum of response from all curated peptides in each protein was used for protein level quantification. The intensity of the proteins was then normalized to the total ion intensity of each sample, with the ratio of protein representing the protein level in each sample.

### 2.5 Cholesterol efflux assay

In order to determine whether changes in the HDL‐bound proteins of interest are associated with changes in the HDL function, we measured the CEC of HDL as previously described by our group.^3^ In summary, the HDL‐CEC assays were performed using the murine macrophage cell line, J774, as per published methods.^17^, ^18^, ^19^ Briefly, 3 x 105 J774 cells/well were plated and radiolabeled for 24 h with 2 µCi of ^3^H‐cholesterol/ml. ATP‐binding cassette transporter A1 (ABCA1) was up‐regulated by incubation with 0.3 mmol/L 8‐(4‐chlorophenylthio)‐cAMP for 16‐hours. ApoB‐depleted serum (2.8%) from participants was added to the efflux medium for 4 h. Liquid scintillation counting was then added to quantify the efflux of radioactive cholesterol from the cells. Efflux was calculated using the standard formula: (µCi of ^3^H‐cholesterol in media containing 2.8% apoB‐depleted participant plasma‐µCi of ^3^H‐cholesterol in plasma‐free media / µCi of ^3^H‐cholesterol in media containing 2.8% apoB‐depleted pooled control plasma‐µCi of ^3^H‐cholesterol in pooled control plasma‐free media). The pooled plasma control was obtained from five healthy adult volunteers and all assays were performed in duplicates run at the same time.

### 2.6 Statistical analysis

The distributions of all variables were examined prior to analysis. Descriptive statistics reported include frequencies and percentages for categorical variables, means and standard deviations for normally distributed continuous variables, and medians and percentiles for non‐normally distributed continuous variables. To compare groups, either the chi‐square test or Fisher's exact test was used for categorical variables, and either the Mann‐Whitney test or *t*‐tests were used for continuous variables. For the initial identification of any proteins that changed significantly after 6 months of treatment (among a total of 84 HDL proteins), we used the Mann‐Whitney test to compare the % change in each protein in the metformin vs. the placebo group. For further analysis of the selected proteins, we performed *T*‐tests on ion intensity values test to confirm our results. Finally, partial least squares discriminant analysis (PLS‐DA) with leave‐one‐out cross‐validation was used to examine whether participants in the two treatment groups could be discriminated on the basis of changes in the proteins. All analyses were performed using R version 3.5.1 or GraphPad Prism version 8.3.0.

## 3 RESULTS

### 3.1 Study population

The participants in the two treatment groups did not differ in age, sex, or race and all were Tanner stage V at baseline (Table 1). Baseline (Table 2) and after 6 months (Table 3) HbA1c, plasma lipids, % body fat and BMI were not significantly different between groups. In a univariate analysis, the insulin dose was significantly different between patients with T1D and HC (Tables 2 and 3).

**TABLE 1:** Demographics of participants treated with metformin or placebo

	Metformin (*n* = 25)	Placebo (*n* = 10)	*p* Value
Age (years)	15.6 ± 1.6	15.5 ± 1.7	.87
Sex n (%)			
Female	19 (76%)	7 (70%)	.69
Male	6 (24%)	3 (30%)	
Race/ethnicity n (%)			
Hispanic	3 (12%)	1 (10%)	1
Non‐hispanic white	20 (80%)	8 (80%)	
Other	2 (8%)	1 (10%)	

Data displayed are mean ± SD or n (%).

**TABLE 2:** The biochemical and clinical variables at baseline by group

	Metformin (*n* = 25)	Placebo (*n* = 10)	*p* Value
	Baseline	Baseline	
HbA1c (%)	9.1 (8.4, 9.4)	8.9 (8.7, 9.1)	1
LDL‐C (mg/dl)	112 (93, 128)	97 (96, 112)	.6479
VLDL‐C (mg/dl)	15 (10.75, 21.75)	22 (16, 26.25)	.0783
HDL‐C (mg/dl)	52 (44, 56)	49 (45, 59.25)	1
Triglyceride (mg/dl)	76 (56, 106)	110 (79, 132)	.0828
Total cholesterol (mg/dl)	178 (157, 206)	174 (167.3, 201.5)	1
DEXA (% body fat)	40 (36, 45)	39 (36, 42)	.9376
BMI (kg/m^2^)	30.1 (27.6, 33.0)	28.7 (26.9, 31.8)	.3034
BMI (percentile)	97.4 (94.5, 98.1)	97.0 (93.0, 97.4)	.3771
BMI Z‐score	1.94 (1.60, 2.08)	1.88 (1.48, 1.94)	.3807
DEXA % fat	40 (36, 45)	39 (36, 42)	.9376
Waist circumference(cm)	94.9 (88.8, 104.0)	95.3 (91.0, 101.9)	.9273
Insulin dose (units/kg/day)	0.96 (0.85, 1.05)	1.21 (1.03, 1.38)	.0207

Note

Values are reported as median (25th %ile, 75th %ile). *p*‐values were calculated using the Mann‐Whitney test.

**TABLE 3:** The biochemical and clinical variables at 6 months by group

	Metformin (*n* = 25)	Placebo (*n* = 10)	*p* Value
	6 months	6 months	
HbA1c (%)	8.8 (8.0, 9.4)	9.0 (8.3, 9.9)	.3235
LDL‐C (mg/dl)	113 (90, 122)	100 (92, 114)	.6919
VLDL‐C (mg/dl)	14.5 (11.0, 20.0)	17.5 (12.0, 20.0)	.4591
HDL‐C (mg/dl)	53.0 (44.0, 60.0)	47.5 (41.0, 56.8)	.3594
Triglyceride (mg/dl)	71.0 (54.8, 99.0)	88.5 (61.8, 98.0)	.4609
Total cholesterol (mg/dl)	179 (160, 193)	172 (157, 188)	.5838
DEXA (% body fat)	41 (37, 44)	40 (35, 43)	.6698
BMI (kg/m^2)^	30.1 (26.9, 32.7)	29.0 (26.6, 32.0)	.6275
BMI (percentile)	96.1 (94.5, 98.1)	95.7 (93.8, 97.8)	.7060
BMI z	1.76 (1.60, 2.07)	1.73 (1.55, 2.01)	.7149
DEXA % fat	41 (37, 44)	40 (35, 43)	.6698
Waist circumference(cm)	93.5 (88.9, 101.5)	92 (82.3, 105.3)	.7150
Insulin dose (units/kg/day)	0.82 (0.68, 0.95)	1.12 (0.92, 1.28)	<.001

Note

Values are reported as median (25^th^ %ile, 75^th^ %ile). *p*‐values were calculated using the Mann‐Whitney test.

### 3.2 Effect of metformin on the HDL proteome

**FIGURE 1:**
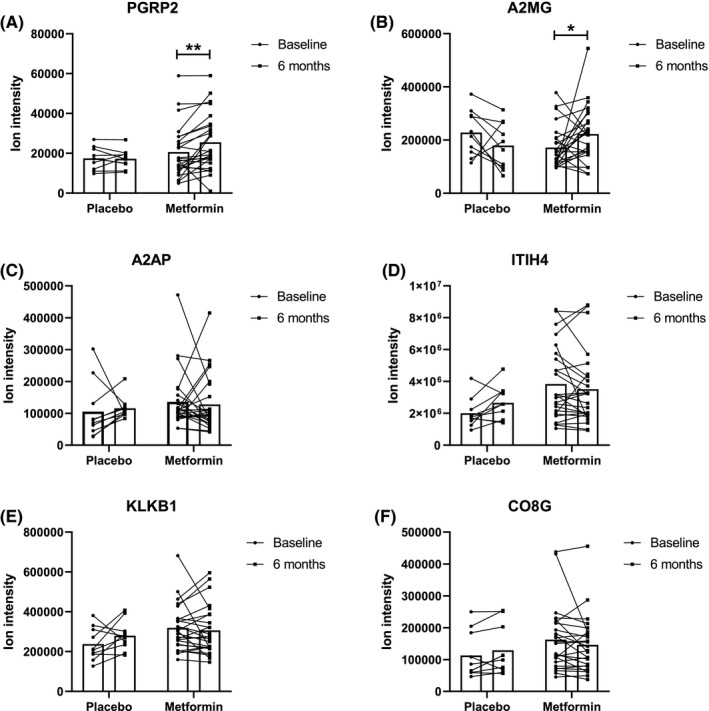
Effect of 6‐month placebo or metformin treatment on HDL‐associated proteins. Ion intensity values detected by data‐independent acquisition (DIA) mass spectrometry analysis of size‐exclusion chromatography purified HDL were compared for subjects receiving placebo or metformin for 6 months. *T*‐tests were used for statistical comparison between baseline and 6‐month time points within each treatment group. * indicates *p* < .05; ** indicates *p* < .01; no indicator is present where comparisons are not statistically significant. A2MG: alpha‐2‐macroglobulin, ITIH4: inter‐alpha‐trypsin inhibitor heavy chain family member 4, KLKB1: kallikrein B1, A2AP: alpha‐2‐antiplasmin, PGRP2: peptidoglycan‐recognition protein 2, CO8: complement 8

**FIGURE 2:**
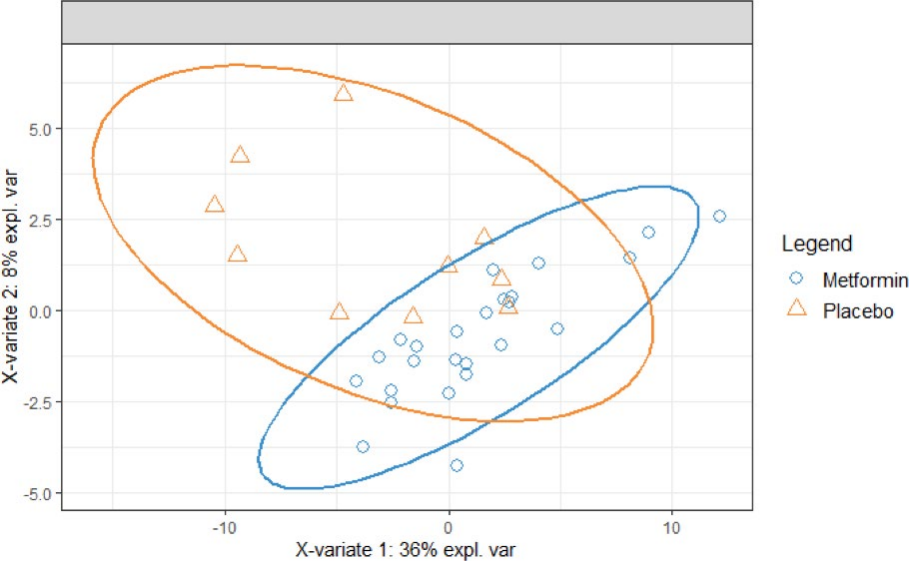
Plot from PLS‐DA for protein changes by treatment group. Partial least squares discriminant analysis (PLS‐DA) was used to determine if the cumulative effects of metformin on the HDL proteome could be used to distinguish treatment groups. Mass spectrometry ion intensities from all 84 quantified proteins were used as input variables for the model. Rankings of the top 10 weighted proteins from components 1 and 2 are listed in Table S2

Using a label‐free SWATH mass spectrometry analysis, an HDL proteome quantification library was generated using a subset of HDL samples. This library allowed for reliable detection of the relative abundance of 84 different HDL‐associated proteins. The Mann–Whitney test was used to screen measured percent changes in protein abundance among treatment groups to identify likely affected proteins (Table S1). This initial screen‐detected differences in average percent change between placebo and metformin groups for six proteins: A2MG (Alpha‐2‐macroglobulin), KLKB1 (Kallikrein B1), ITIH4 (Inter‐alpha‐trypsin inhibitor heavy chain H4), A2AP (Alpha 2‐antiplasmin), PGRP2 (Peptidoglycan recognition protein), CO8G (Complement C8 Gamma Chain). These candidate proteins were then subjected to statistical analysis of raw ion intensity measurements by *T*‐test. With this analysis, only PGRP2 (+23.4%, *p* = .0058) and A2MG and alpha‐2‐macroglobulin (+29.8%, *p* = .049) displayed statistically significant increases in ion intensity in response to metformin treatment (Figure 1A,B) while no effect was seen for placebo. The other four proteins were not significantly affected by metformin when analysed this way (Figure 1C‐F). To determine if the cumulative minor effects of metformin on the HDL proteome could discriminate between treatment groups, partial least squares discriminant analysis (PLS‐DA) was performed. Discrimination of the treatment groups by changes in proteins was minimal (Figure 2) and the ROC AUC for this model was 0.6, only slightly better than chance. In this analysis, A2MG contributed most to discrimination between treatment groups (Table S2). In our analysis, we had identified one outlier with a protein that could skew the data. We also performed sensitivity analysis which indicated that removal of the outlier point did not impact the ultimate conclusion of the statistical comparison.

### 3.3 Effect of metformin on CEC

**FIGURE 3:**
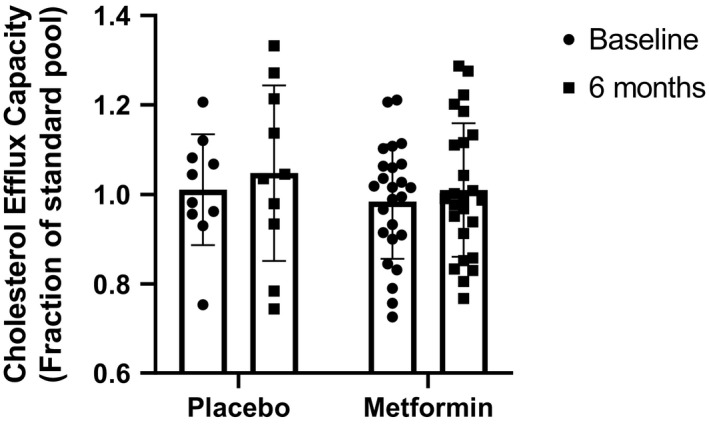
Effect of placebo or metformin on cholesterol efflux capacity. Cholesterol efflux to apoB‐depleted serum was measured using cAMP‐treated J774 macrophage cells loaded with radiolabeled cholesterol. Efflux was normalized to a healthy control sample pool. Data were analysed using repeated measures two‐way ANOVA with Sidak correction for multiple comparisons. No indicator is present where comparisons are not statistically significant

To evaluate the possible impact of metformin on the most studied function of HDL, CEC was measured in all participants at baseline and 6 months follow‐up. CEC of apoB‐depleted plasma from each participant was measured using cholesterol‐loaded J774 macrophages stimulated with cAMP to upregulate ABCA1 expression. There was no significant difference between treatment groups at baseline or after 6 months of treatment with metformin or placebo (Figure 3).

## 4 DISCUSSION

In this analysis, from age and sex‐matched youth with T1D treated with metformin or placebo for 6 months, we demonstrate for the first time that metformin has an increase on the PRGP2 (peptidoglycan recognition protein 2 also known as N‐acetylmuramoyl‐L‐alanine amidase) and A2MG (Alpha‐2‐macroglobulin) proteins on HDL. The effect of metformin on PGRP2 and A2MG could potentially influence HDL function and impact the cardiovascular health of T1D patients receiving this treatment. In this sub‐study, we did not perform further mechanistic experiments to understand how functions of HDL might be affected by enrichment of the HDL molecule with the PGRP2 and A2MG protein. However, we do demonstrate that it does not appear to involve cholesterol efflux activity, the most widely studied HDL function. While further experiments are needed to fully elucidate the mechanisms by which metformin alters PGRP2 and A2MG protein on HDL and how this may impact HDL function, we discuss below how our findings relate to what is known about HDL‐associated PRGP2 and A2MG and how this protein might contribute to CVD risk.

In the current study, we found that HDL‐associated PGRP2 increased significantly after metformin treatment while no change was observed in the placebo group. Our group has previously published that PGRP2 was higher in the HDL of youth with T1D compared to healthy controls.^20^ This would suggest that the effect of metformin shifts the level of this protein even further from the level of healthy control participants rather than normalizing it toward the level of healthy controls. We also have shown previously that PGRP2 was not associated with glycemic control in youth with T1D.^20^ It is possible that the percent change in PRGP2 may be more closely related to other factors relative to the metformin use rather than glycemic control. In fact, the T1D‐Metformin RCT trial did not show any significant impact of metformin on HbA1c.^11^ Overall, our findings suggest that metformin increases the PGRP2 and A2MG proteins on HDL and below we discuss the possible effects on metabolic and cardiovascular health based on what is known so far about the function of these proteins.

Interestingly, one study found that patients with the acute coronary syndrome had lower expression of PGRP2 levels in HDL compared to patients with stable coronary disease, even though no difference was found when compared to HDL levels of healthy controls.^21^ These patients were older men, with and without coronary disease and only 1/30 participants had diabetes. It is likely that age, sex and diabetes status might further explain alterations of PRGP2 levels in HDL. Also, the peptidoglycan recognition protein 1, (PGRP1), which belongs to the same family of proteins, was found to be associated with atherosclerotic cardiovascular events in the Dallas Heart Study and participants with the highest levels of PGRP1 were >3 times more likely to develop CVD.^22^

Peptidoglycan recognition proteins are known to be responsible for keeping a normal gut microbiome environment and protect the host from inflammation and colitis.^23^ PGRP2 is present in the serum, the skin and the epithelial cells of the intestines.^24^ The expression of PGRP2 is highest in the liver, from where it is secreted in the blood, and PGRP2 expression is also induced by bacteria in the epithelial cells of all segments of the gastrointestinal tract.^23^ There is growing evidence about the role of altered gut microbiota to promote CVD via several mechanisms, such as altering lipid metabolism, playing a role in vascular dysfunction and hypertension, increasing systemic inflammation, foam cell formation and insulin resistance.^25^ There is recent evidence that treatment with metformin not only alters the gut microbiome but some of the beneficial effects of metformin could be due to the change in the gut microbiome.^26^, ^27^ It is also known that the intestine has a significant contribution to the production of HDL‐C.^28^ Based on the above, it is possible that metformin alterations to the gut microbiome could be associated with changes in the peptidoglycan protein expression of PRGP2 in the intestines and also on PRGP2 concentration on HDL.

We also found that A2MG increased after metformin. Our group has previously found that the A2MG protein of HDL was positively associated with calcified burden of coronary angiography, a marker of increased CVD risk.^29^ Another group also found higher A2MG protein of HDL in patients with non‐alcoholic fatty liver disease, suggestive of increased fibrinogenesis activity.^30^ More research is needed to explore whether metformin changes the anti‐thrombotic function of HDL by altering the expression of A2MG protein.

Our study did not find any effects of metformin on HDL efflux capacity, contrary to our hypothesis. A previous study by Matsuki et al^31^ showed that glycation of HDL reduced the CEC of HDL and that metformin restored this defect in studies done *in vitro*, by preventing the glycation of HDL. One possible explanation for the difference with our findings is that the dose used for the in vitro studies was relatively higher and had a more potent effect than the dose that has been approved for use in humans. In addition, in our study, all patients had diabetes with a HbA1c well above target, and we thus assume that a certain degree of glycation of HDL was present in all of them. However, our study was not designed to look at the glycated forms of HDL. It is possible that the effect of metformin in restoring CEC might be different in certain patient populations with a high degree of glycated HDL. Future studies can examine the factors that affect the glycation of proteins of HDL in patients with T1D and whether metformin or other diabetes drugs such as insulin could restore the CEC of glycated forms of HDL in patients with T1D. Further studies are needed to examine the exact role of PRGP2 and A2MG on HDL function and whether metformin's effect on gut microbiota affects the expression of PGRP2 in the intestine and HDL.

Of note, 3 of the 6 proteins on our initial screening are involved in the coagulation pathway and formation of the fibrin clot: A2MG, alpha‐2‐macroglobulin, KLKBI, Kallikrein B1, and A2AP, Alpha 2‐antiplasmin. These pathways are involved in the pathogenesis of atherosclerosis and coagulation, which supports the hypothesis that changes in these proteins could affect the anti‐thrombotic HDL function.^32^ Our group had also previously found that KLKB1 of HDL was associated with lower CEC.^29^ However, given that KLKBI and A2AP proteins also increased in some participants in the placebo group (Figure 1), we believe more research is needed to explore whether metformin changes the anti‐thrombotic function of HDL by altering the expression of the above proteins.

Strengths of our study include the fact that we were able to match participants that were treated with metformin or placebo in a double‐blinded randomized fashion. All participants were overweight and had similar glycemic control and pubertal stage. We also have detailed HDL proteome data: The two‐step size‐exclusion chromatography and lipid interaction‐based HDL purification and the SWATH‐MS allowed us to have a very sensitive and robust label‐free proteomic quantitation for multiple clinical samples. Our two‐step approach to the proteomics data analysis included an initial screening for metformin affected proteins based on percent change by treatment group and was followed up with direct comparisons of candidate proteins by their detected ion intensities. This approach improved confidence in detected differences and reduced the false discovery rate by filtering out weaker effects. Limitations of this study include our small sample size and the lack of additional HDL function assays or assays directly related to PGRP2 function and the lack of directly measured insulin sensitivity. A limitation of our HDL purification method is that it does not allow for analysis of HDL subfractions which are commonly characterized by density and isolated by ultracentrifugation.^33^ One advantage of our approach over ultracentrifugation is that it purifies HDL under physiological g forces and salt conditions resulting in less disruption of protein interactions on lipoproteins caused by extreme conditions experienced during ultracentrifugation.^34^, ^35^ The downside is that size‐exclusion chromatography by itself does not isolate pure HDL preparations and there is some contamination of non‐lipoprotein‐associated proteins (e.g. albumin and immunoglobulins). This is overcome by our two‐step isolation, which includes washing of lipoproteins on a lipid‐binding resin.^13^ Alternative approaches to the isolation of native HDL include immunoaffinity adsorption usually by pulldown with anti‐apolipoprotein A‐I antibodies.^36^, ^37^ This technique produces clean HDL, but it is not known if some HDL‐binding proteins can influence antibody interactions with HDL and this approach may exclude lipoproteins in the HDL size range which do not contain apolipoprotein A‐I. However, the goal for this pilot project was to provide an initial characterization of the effect of metformin on the proteomic composition of HDL and identify associations with changes in cholesterol efflux function. Even though we did not find a significant change in CEC, it is possible this was due to our relatively small sample size or that the treatment did not have an impact on pre‐beta HDL, the particle species predominantly responsible for ABCA1‐mediated cholesterol efflux.^38^ Pre‐beta HDL are small discoidal particles composed of apolipoprotein A‐I, phospholipid, and free cholesterol, and they do not contain many other proteins.^39^ Therefore, effects observed in the HDL proteome are likely not reflective of changes to pre‐beta particles. Although it is possible that redistribution of apoA‐I or other proteins among HDL subspecies could occur even if no change in the total apoA‐I protein was observed. However, this could not be detected without isolation of distinct HDL subfractions. It is also possible that there may be interesting glycation modifications to HDL proteins that could impact particle functions and might be prevented or reversed by metformin, but we were unable to examine these in the present analysis. Future studies can further explore whether enrichment of HDL with PRGP2 could potentially alter HDL function.

In summary, we found a significant increase in the PGRP2 and A2MG content of HDL in youth with T1D treated with metformin compared to placebo by combining size‐exclusion chromatography‐based HDL purification and SWATH‐MS‐based label‐free proteomic quantitation. Further studies are needed to explore whether this effect of metformin impacts other known functions of HDL such as the anti‐inflammatory or anticoagulant activity, and to also explore potential novel functions arising from this interaction. With the recent rapid advancements in proteomics technologies, future studies to investigate the factors that contribute to the glycation of HDL proteins in patients with T1D and how those are impacted by metformin or other diabetes drugs will be of significant interest.

## CONFLICT OF INTEREST

Authors have nothing to disclose.

## AUTHOR CONTRIBUTIONS

Evgenia Gourgari: Conceptualization, development of the project, acquired funding for the project, helped with the experiments, and wrote the initial manuscript. Kristen J. Nadeau: Assisted in collection of clinical data from the registry, reviewed and edited the manuscript. Laura Pyle: Did the statistical analysis, reviewed and edited the manuscript. Martin P. Playford: Did the cholesterol efflux experiments, reviewed and edited the manuscript. Junfeng Ma: Did the proteomics experiments, reviewed and edited the manuscript. Nehal N. Mehta: Reviewed and edited the manuscript. Alan T. Remaley: Conceptualization, development of the project, reviewed and edited the manuscript. Scott M. Gordon: Conceptualization, development of the project, did the proteomics experiments, reviewed, edited and approved the final manuscript.

## PATIENT CONSENT STATEMENT

Patients gave consent to participate in the T1D‐Exchange Metformin RCT.

## Supporting information

Table S1‐S2

## Data Availability

Not applicable.
