# Supplementary material for: Effect of metformin on the high‐density lipoprotein proteome in youth with type 1 diabetes
Source: Endocrinol Diabetes Metab. 2021 May 9;4(3):e00261. doi: 10.1002/edm2.261 (PMC8279605; doi:10.1002/edm2.261)
Supplement: Supplementary file 1 — Table S1‐S2 [file EDM2-4-e00261-s001.docx]

**Supplemental Data**
